# Customized Tracheostomy Cannula as a Therapeutic Adjunct in Tracheal Stenosis

**DOI:** 10.1155/2013/921365

**Published:** 2013-04-03

**Authors:** Doh Young Lee, Jungirl Seok, Wonjae Cha, Won Yong Lee, J. Hun. Hah, Tack-Kyun Kwon, Kwang Hyun Kim, Myung-Whun Sung

**Affiliations:** Department of Otorhinolaryngology, Head and Neck Surgery, Seoul National University, College of Medicine, Seoul National University Hospital, 101 Daehak-ro, Jongno-gu, Seoul 110-744, Republic of Korea

## Abstract

Tracheotomy is often successfully used to manage tracheal stenosis, as a temporizing measure prior to definitive treatment or a long-term remedy. In some patients, where a sizeable portion trachea is stenotic, the fixed arm of an ordinary tracheotomy tube may not be of sufficient length to satisfactorily maintain the distal tracheal lumen, and commercially available adjustable tubes may not be at hand in certain clinical settings. Herein, we describe a simple method of constructing a temporary tracheotomy tube with an adjustable distal arm, allowing custom fit at the patient bedside.

## 1. Introduction

Tracheal stenosis is a serious and potentially life-threatening condition of varied etiology [1, 2]. While tracheotomy usually maintains the airway prior to definitive treatment and may even serve as a long-term solution in some patients, the fixed distal arms of regular tracheotomy tubes simply do not provide the support needed to maintain tracheal lumen when the stenotic segment is lengthy. Furthermore, the tips of such relatively short tubes may be a source of mucosal irritation to exacerbate a patient's condition. For airway stenting of either temporary or long-term duration, the stent tip ideally should bypass the stenosis without sacrificing ventilation to either of the lungs. Severe stenosis of the distal trachea thus poses a distinct challenge to surgeons who render care.

## 2. Case Presentation

 A 17-year-old male was referred to our institution for airway stenosis. He had been injured while bicycling (traffic collision), undergoing intensive treatment elsewhere for subarachnoid hemorrhage, left mandibular fracture, a fractured clavicle, and bilateral pneumothoraces. Tracheotomy was subsequently performed to accommodate long-term mechanical ventilation. In a matter of two weeks, however, he developed inspiratory stridor and respiratory distress due to obstructive proliferation of granulation tissue at the cannula tip. An endotracheal tube with internal diameter of 6 mm was inserted for temporary relief, but the problem recurred at the tip of the endotracheal tube.

**Figure 1:**
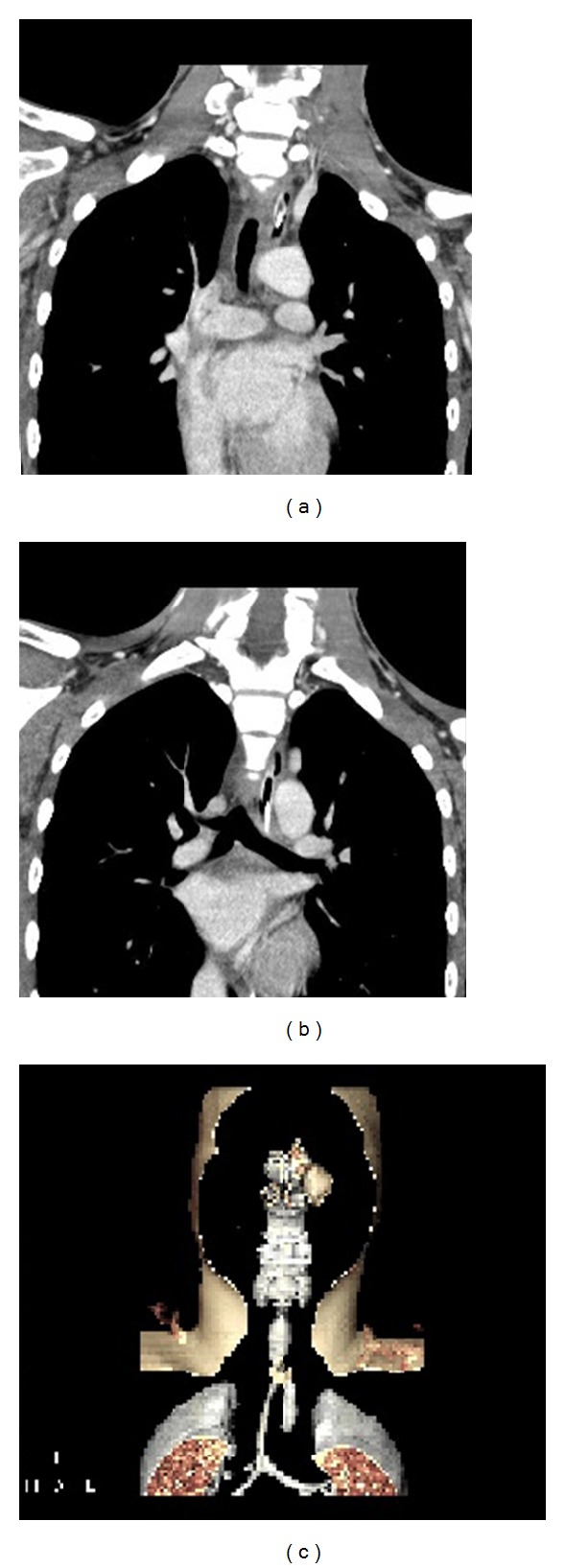
Initial CT of airway in coronal view with narrowed tracheal lumen of 1 cm (a) and congenital loss of right main bronchus (b), reconstructed in 3D (c).

**Figure 2:**
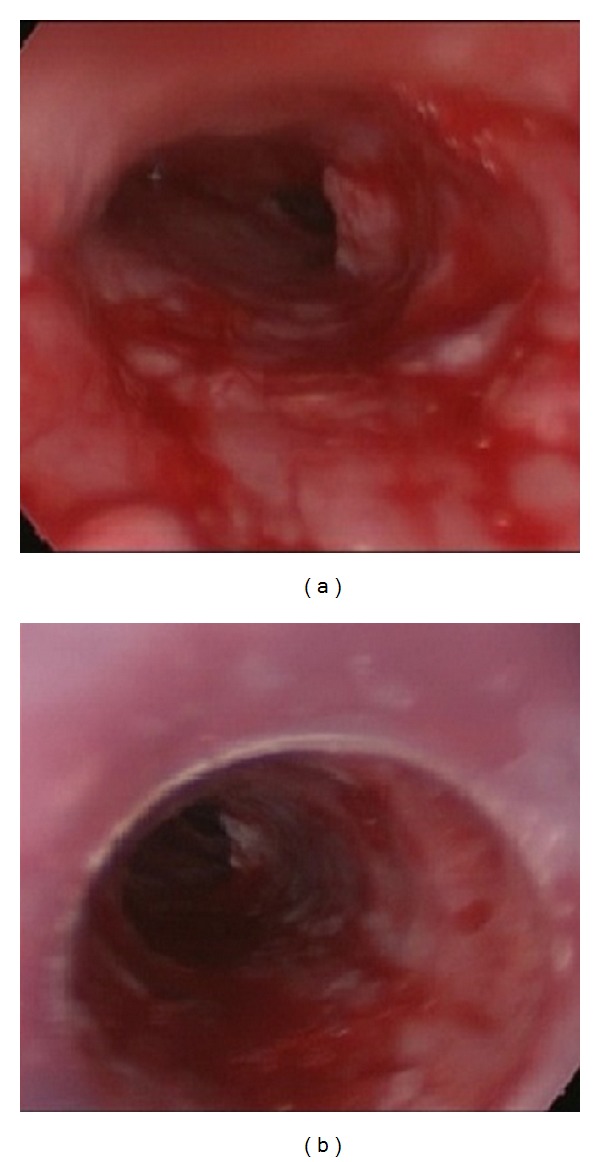
Severely inflamed mucosa with bleeding to touch (a). After insertion of Montgomery T-tube, second procedure (b).

At this point, the patient was referred to our facility. Computed tomography (CT) showed narrowing of the entire trachea (Figure 1), prompting evaluation by rigid bronchoscopy. Upon endotracheal tube removal, there was copious oozing of blood from eroded and inflamed tracheal mucosa (Figure 2(a)). Nevertheless, a short silicone T-tube 4.5 cm (the length of lower arm) kept the narrowed and severe inflamed lumen widely patent.

On postoperative day 5, the patient abruptly became dyspneic, developing labored breathing with inspiratory stridor and visible chest retraction. His respirations exceeded 40/min. Fiberoptic endoscopy confirmed significant proliferation of granulation tissue at and distal to the T-tube tip. With respiratory failure imminent, the T-tube was replaced with a long E-tube using a stylet at bedside. Thereafter, we obliterated the newly-formed granulation tissue using cold instruments and laser during rigid bronchoscopy. An endotracheal tube of 8.5 cm (the length of distal arm) was prepared intraoperatively and successfully placed in the trachea, entirely bypassing the inflamed stenotic segment. Required tubal length was gauged under bronchoscope guidance, and the final outer diameter (1.0 cm) was determined through sequential bougienation trials.

Several days later, dyspnea returned, again as a consequence of granulation tissue at the cannula tip which was confirmed by fiberoscopic examination. This time, a longer, customized tracheostomy cannula of 9 cm was inserted at bedside to definitively bypass the stenosis. When a fourth and final bronchoscopy was performed in another two weeks, we saw complete resolution of prior complications, marked by clearing of inflammation, absence of granulation tissue, and a patent airway (Figure 2(b)). The customized tracheostomy cannula was then replaced by a Montgomery T-tube, longer in distal reach (7.8 cm) than the previous one (4.5 cm). Three weeks later, this patient was symptom-free and was discharged for home care. He will be monitored in the next several months for containment of mucosal inflammation and proper maturation of the tracheal framework.

## 3. Method for Preparing a Customized Tracheostomy Cannula from Endotracheal Tubes

**Figure 3:**
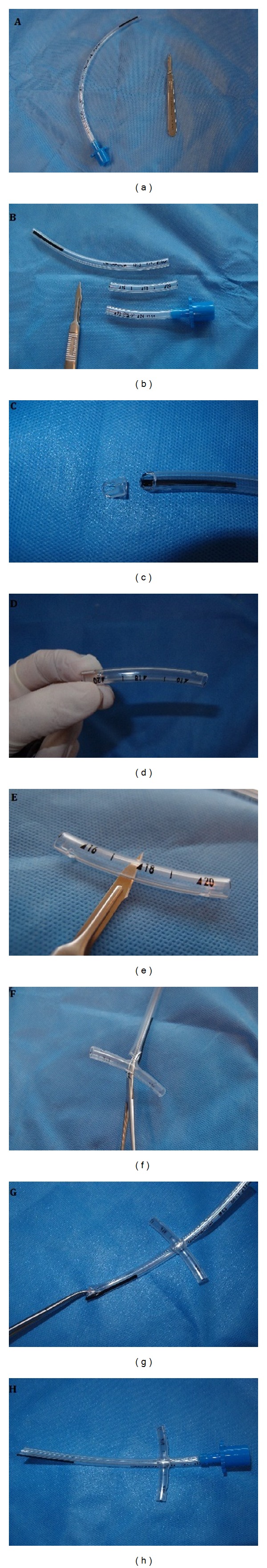
Customized tracheostomy cannula. Using 1 E-tube and blade (a), divide the E-tube into 3 segments (b), longest one is for maintaining the airway, and the other is used for stent fixation. The beveled tip and side hole are removed. (c) Two holes are placed at opposing sides of upper end for string attachment. (d) Two holes are then made (front and back) at mid-section, allowing the curved tube to approximate the patient's neck contours. (e) The airway tube is ultimately inserted and fixed, leaving an appropriate length distally and a 5 cm excess at the proximal end. (f), (g) Both tubes (airway and fixation) together are finally inserted and secured with nylon suture (2.0 or 3.0) (h).

We generally use one plain endotracheal tube to construct a customized tracheostomy cannula and start by assessing a degree of stenosis with a rigid bronchoscope. This enables us to gauge the size of stent needed. The endotracheal tube is cut to a length of about 10 cm to maintain the airway, with remainder used for stent fixation. Two holes are then made (front and back) at mid-section, allowing the curved tube to approximate the patient's neck contours. Another two holes are placed at opposing sides of its upper end for string attachment. The airway tube is ultimately inserted and fixed, leaving an appropriate length distally and a 5 cm excess at the proximal end. Both tubes (airway and fixation) together are finally combined and secured with nylon suture (2.0 or 3.0). Optimal positioning of the customized tracheostomy cannula tip should be confirmed with a fiberoptic scope, before the neck string is fastened (Figure 3).

## 4. Discussion

Surgical resection with reanastomosis is a reasonable strategy, when tracheal stenosis is limited and confined to proximal trachea [3]. With proper patient selection, this approach has a high rate of decannulation (>90%) [4, 5]. In patients who are otherwise poor surgical candidates, whether by site or extent of stenosis, treatment is generally relegated to bougienation, laser ablation, and stent insertion [6, 7].

While a number of surgical methods for stent insertion have met with success, tracheal stents are typically plagued by recurrent stenosis or obstruction due to granulation tissue [8]. When this occurs, a long endotracheal tube may suffice to relieve the impasse and maintain air flow in a crisis, but emergency surgery is usually the only recourse when distal trachea is blocked. For difficult insertions, endotracheal tube insertion may require a stylet or fiberoptic guidance, in which case there is a chance of further injury to tracheal mucosa, exacerbating the tissue reaction. This is a fairly risky procedure, requiring an experienced physician, and should be done only as a temporizing measure for eventual surgery.

One explanation for the problematic granulation tissue encountered is the slight yet relentless repositioning of the stent in the course of neck movement. Repetitive mucosal irritation of this nature, however minor, encourages a reactive response; and with the complete cartilaginous ring of tracheal stenosis, no soft posterior membrane exists to absorb this type of trauma. A customized tracheostomy cannula is of benefit in this regard by enabling optimal positioning of the stent tip and secure anchoring to skin at the tracheostomy site. Stent mobility and potential traumatic injury to tracheal mucosa are thus minimized. The fixation arm is also easily sutured, greater stability should be required. Above advantages underscore the superiority of a customized tracheostomy cannula over a conventional endotracheal tube or established customized cannula in this setting [9–11].

Based on our firsthand experience with stent-related restenosis, we have come to rely on our own version of tracheal cannula. It is adjustable, versatile, and easily reproduced (even at bedside as shown). For high-risk patients, it can be sized/prepped and sterilized with ease. Of note, we routinely remove the beveled tip and side holes of a conventional E-tube when fashioning a customized tracheostomy cannula. There is a tendency for granulation tissue to grow through the side hole, so the entire beveled end (with side holes) is removed and smoothed out. 

It should be emphasized as well that a customized cannula is not a permanent solution to the patients with long tracheal stenosis. However, by simple designing and preparation, we can make an excellent customization of the distal length of the tracheotomy tube which will be fit for the patient.
